# Drivers of delayed care-seeking among children aged 6–35 months with medically-attended diarrhea: Findings from the Enterics for Global Health (EFGH)-Shigella surveillance study, 2022–2024

**DOI:** 10.1371/journal.pgph.0007433

**Published:** 2026-11-13

**Authors:** Maureen Ndalama, Olivia Schultes, Thomasena O’Byrne, Patricia Pavlinac, Beth A. Tippett Barr, Sonia I. Rao, Sean R. Galagan, Akello Isaiah, Stephen Munga, Adama Mamby Keita, Jahangir Hossain, Mehrab Karim, Desiree Witte, Jennifer Cornick, Farhana Khanam, Farah Qamar, Zubair Latif, Naveed Ahmed, Sadia Islam, Maribel Parades, Richard Omore, Kirkby D. Tickell

**Affiliations:** 1 Malawi Liverpool Wellcome Research Programme, Blantyre, Malawi; 2 Department of Global Health, University of Washington, Seattle, Washington, United States of America; 3 Liverpool School of Tropical Medicine, Liverpool, United Kingdom; 4 Department of Epidemiology, University of Washington, Seattle, Washington, United States of America; 5 Nyanja Health Research Institute, Salima, Malawi; 6 Centre for Global Health Research, Kenya Medical Research Institute, Kisumu, Kenya; 7 Medical Research Council Unit, The Gambia at the London School of Hygiene and Tropical medicine, Banjul, The Gambia; 8 Institute of Infection, Veterinary and Ecological Sciences, Faculty of Health and Life Sciences, University of Liverpool, Liverpool, United Kingdom; 9 Infectious Diseases Division, International Centre for Diarrhea Disease Research, Dhaka, Bangladesh; 10 Department of Pediatrics and Child Health, The Aga Khan University, Karachi, Pakistan; 11 Asociación Benéfica Prisma, Unidad de Investigaciones Biomedicas, Iquitos, Peru; PLOS: Public Library of Science, UNITED STATES OF AMERICA

## Abstract

Timely healthcare seeking is critical to reducing preventable deaths from childhood diarrhea. We examined host, clinical, and sociodemographic factors associated with delayed seeking for medically-attended diarrhea (MAD) among children aged 6–35 months in the Enterics for Global Health (EFGH) study. We conducted a cross-sectional analysis among children aged 6–35 months enrolled in the EFGH study with MAD from Bangladesh, The Gambia, Kenya, Malawi, Mali, Pakistan, and Peru. Delayed care seeking was defined as presenting to a study facility ≥2 days after diarrhea onset; a sensitivity analysis used ≥4 days. Prevalence of delayed care-seeking was 61.7% across study sites. Delays were common among younger children, younger caregivers, those who sought prior care, and those presenting with severe illness. Children aged 12–23 months (aPR = 0.96; 95% CI: 0.93–0.99) and 24–35 months (aPR = 0.90; 95% CI: 0.86–0.94) were less likely to experience delays compared to those aged 6–11 months. Caregivers aged 20–29 years (aPR = 0.92; 95% CI: 0.88–0.96) and 30–39 years (aPR = 0.91; 95% CI: 0.86–0.95) were also less likely to delay compared to caregivers under 20 years. Prior care-seeking was strongly associated with delay, including visits to medical providers (aPR = 1.29; 95% CI: 1.23–1.35), community health workers (aPR = 1.25; 95% CI: 1.13–1.38), pharmacies (aPR = 1.21; 95% CI: 1.15–1.27), and traditional healers (aPR = 1.29; 95% CI: 1.19–1.40). Indicators of clinical severity were also linked to delay, including some dehydration (aPR = 1.08; 95% CI: 1.03–1.13), dysentery (aPR = 1.15; 95% CI: 1.11–1.20), and severe Modified Vesikari Score (aPR = 1.13; 95% CI: 1.09–1.18). Delayed healthcare seeking for childhood diarrhea is common in high-burden LMIC settings and varies substantially by country.

## Introduction

Delay in healthcare seeking for a child with acute diarrhea has the potential risk of increasing chance for disease severity, preventable hospitalization due to life threatening complications and subsequent death [1]. Although several studies have attempted to evaluate drivers and patterns of healthcare seeking among caregivers of young children with medically-attended diarrhea (MAD) in low and middle-income countries (LMICs), variations have been observed likely due to methodological deficiencies; including studies conducted at single site or used non-standardized protocols across multiple sites which consequently limits the utility of data from such studies to inform more impactful regional policy decisions [2].

The Enterics for Global Health (EFGH)-*Shigella* surveillance study is a two-year surveillance study with a primary aim of determining the incidence of *Shigella*-attributed MAD in children 6–35 months of age living in populations whose demographic characteristics are well described from seven LMICs representing high disease burden settings in South America, South East Asia and sub-Saharan Africa as described elsewhere [3].

Knowledge of factors determining healthcare seeking is important for the improvements in child survival and care seeking interventions against childhood diarrheal illness, especially in high disease burden settings such as EFGH sites. Addressing these barriers could help reduce preventable child deaths and contribute to achieving Sustainable Development Goal 3 which aims to ensure healthy lives and promote well-being for all at all ages, including reducing preventable deaths among children under five years.

The current project is nested in the EFGH-*Shigella* surveillance study and aims to identify host, clinical and sociodemographic factors associated with delay in healthcare seeking behavior for diarrheal illness among children aged 6–35 months residing from the EFGH sites located in Peru, Pakistan, Bangladesh, Malawi, Mali, The Gambia, and Kenya.

## Methods

### Ethics statement

The EFGH study was reviewed and approved by respective institutional and ethical review boards at each EFGH site (Kenya: Kenya Medical Research Institute-KEMRI; Malawi: College of Medicine Research Ethics Committee, and University of Liverpool REC.; The Gambia: The Gambia Government/MRC Joint Ethics Committee and London School of Hygiene & Tropical Medicine Ethics Committee; Bangladesh: icddr,b Ethical Review Committee; Pakistan: Aga Khan University Ethical Review Committee, Peru: AB PRISMA Ethics Committee, Mali: Faculté de Médecine, de Pharmacie et d'Odento-Stamologie (FMPOS), the University of Washington, Seattle, USA, The University of Maryland, Baltimore, Baltimore, USA, The University of Virginia, Charlottesville, USA, and Emory University, Atlanta, USA. This study was conducted according to Good Clinical Practice (GCP), including Good Clinical Laboratory Practice (GCLP), the Declaration of Helsinki, IRB and local rules and regulations specific to each EFGH country. Verbal consent was obtained prior to screening. After confirming eligibility, informed written consent was taken from the parent or legal guardian (primary caregiver) present at the EFGH health facility.

### Study design and setting

Participants were recruited from children who presented with diarrhea at selected outpatient and referral health facilities serving defined catchment populations across the seven study countries While facilities varied somewhat by country, all were primary healthcare centers, health centres, district hospitals, or referral facilities that routinely manage pediatric diarrheal illness and served Recruitment and follow-up occurred from 2022 to 2024.

We conducted a nested cross-sectional analysis of enrolled children to identify host, clinical, and sociodemographic factors associated with delayed care seeking for diarrhea among children aged 6–35 months.

### Study participants

A child was included in the study if they were aged 6–35 months and presented to an EFGH sentinel health facility with diarrhea (≥3 abnormally loose or watery stools in the previous 24 hours), diarrhea onset was ≤7 days ago, the primary caregiver and child resided within the study area and planned to remain at their current residence for at least four months, the primary caregiver was able to provide informed consent (legal age or emancipated minor) in a language for which translations were available, and fewer than four hours had passed since the child presented to the health facility to ensure that enrollment procedures, clinical assessments, anthropometry, and specimen collection occurred as close to presentation as possible and before substantial clinical management or treatment could alter measured signs and symptoms.

### Data collection

At enrollment, study staff asked caregivers about sociodemographic characteristics and the child’s clinical history. Staff also performed a clinical examination of the child and measured weight and height. Additional information on symptoms during the index diarrhea episode was captured through a diarrhea diary filled out by the caregiver and returned at the four week or three-month follow-up visit, as well as questions asked at those visits. Staff recorded vaccination history from a written source at enrollment or follow-up visits, if available. Hospitalization was recorded if occurring at enrollment and was otherwise collected at follow-up visits by caregiver recall and hospital records.

### Definitions

The primary outcome variable, delayed care seeking, was defined as attending an EFGH recruitment health facility two or more days after the onset of diarrhea. In sensitivity analyses, very delayed care seeking was defined as four or more days between diarrhea onset and health facility attendance. Dysentery was defined either by caregiver report of blood in the stool at any point during the diarrhea episode or by clinician diagnosis at enrollment. Dehydration status was assessed at enrollment and categorized according to the World Health Organization (WHO) Integrated Management of Childhood Illness (IMCI) guidelines [4].

Nutritional status indicators were based on standard WHO growth measures. Children were categorized as wasted, stunted, or underweight based on weight-for-height z-scores, height-for-age z-scores, and weight-for-age z-scores, respectively. Any z-scores above +6 or below −6 were considered biologically implausible and treated as unknown, in line with WHO guidance [5].

Prior care seeking was defined as the type of care a caregiver consulted before the child’s enrollment in the study. When multiple sources were reported, the participant was assigned to the highest level of care accessed, following this hierarchy: health facility (hospital, emergency room, or pediatrician), community health worker or health outpost, pharmacy or drug seller, traditional or religious healer, other or none. The wealth index was derived from a weighted sum of country-specific indicators adapted from the Demographic and Health Survey (DHS) and designed to reflect within country wealth quintiles [6]. Lastly, age-appropriate vaccination was defined as receipt of all scheduled doses of eight key vaccines; BCG, Polio, DPT, Hepatitis B, Haemophilus influenzae type b, Rotavirus, MMR, and pneumococcal pneumonia based on each country's national immunization schedule, with a one-month grace period allowed for each dose.

### Statistical analysis

Descriptive statistics for predictor variables were presented using medians and interquartile ranges for continuous variables and counts and proportions for categorical variables. To describe the association between host, clinical and sociodemographic factors and delayed care seeking, prevalence ratios (PRs) were estimated using modified Poisson regression with a priori adjustment for study site, labelled using the country where children were enrolled. Models used generalized estimating equations with robust standard errors to account for multiple enrollments of the same child. A sensitivity analysis restricted the dataset to the first enrollment for each child. A second sensitivity analysis was performed where delayed care seeking was defined as seeking care four or more days after diarrhea onset.

In both the primary analysis and the sensitivity analysis, multivariate models included risk factors with a p-value of 0.02 or less and adjusted for study site. All severity indicators were associated with each other by the chi squared test (p < 0.05). To avoid collinearity of severity indicators, dysentery and dehydration (primary analysis) or only dysentery (sensitivity analysis) was included in all multivariate models except for moderate-to-severe diarrhea (GEMS-MSD) and Modified Vesikari Score (MVS). Models examining GEMS-MSD did not include MVS, dysentery or dehydration, while models examining MVS did not include GEMS-MSD, dysentery or dehydration.

We also ran univariate models stratified by enrollment site. Due to smaller sample sizes, we used a binary definition for prior care-seeking (any versus none) and wealth index (least or less wealth versus middle, more, and most wealth). All analyses were conducted in R version 4.3.

## Results

### Sociodemographic characteristics

Among the 9,476 enrolled children, 55% were male and nearly half (46%) were aged 12–23 months (Table 1). 61% of caregivers were aged 20–29 years. Educational attainment showed that 48.3% of the caregivers have attended some secondary school. There were variations in country specific education attainment, in Peru 88% of the caregivers had at least some secondary education, compared to only 18% of the caregivers in The Gambia and 25% of the caregivers in Mali. Attendance at Koranic schools was reported only in 17% of the caregivers in Mali, 2% of caregivers in Pakistan and 33% of the caregivers in The Gambia. Among 8,587 unique children, 773 were enrolled more than once (Table A in S1 Appendix).

**Table 1:** Participant characteristics.

Characteristic	Bangladesh	Kenya	Malawi	Mali	Pakistan	Peru	The Gambia	Overall
	N = 1,361*^1^*	N = 1,400*^1^*	N = 1,399*^1^*	N = 1,400*^1^*	N = 1,400*^1^*	N = 1,117*^1^*	N = 1,399*^1^*	N = 9,476*^1^*
Age (months)								
6-11	572 (42.0%)	560 (40.0%)	478 (34.2%)	600 (42.9%)	454 (32.4%)	365 (32.7%)	446 (31.9%)	3,475 (36.7%)
12-23	588 (43.2%)	595 (42.5%)	627 (44.8%)	613 (43.8%)	646 (46.1%)	585 (52.4%)	718 (51.3%)	4,372 (46.1%)
24-35	201 (14.8%)	245 (17.5%)	294 (21.0%)	187 (13.4%)	300 (21.4%)	167 (15.0%)	235 (16.8%)	1,629 (17.2%)
Sex								
Female	596 (43.8%)	637 (45.5%)	651 (46.5%)	643 (45.9%)	655 (46.8%)	488 (43.7%)	646 (46.2%)	4,316 (45.5%)
Male	765 (56.2%)	763 (54.5%)	748 (53.5%)	757 (54.1%)	745 (53.2%)	629 (56.3%)	753 (53.8%)	5,160 (54.5%)
Maternal education								
Primary school or less	506 (37.2%)	615 (44.0%)	620 (44.3%)	809 (58.3%)	750 (53.6%)	174 (15.6%)	682 (48.8%)	4,156 (43.9%)
Some secondary or greater	855 (62.8%)	782 (56.0%)	779 (55.7%)	343 (24.7%)	616 (44.0%)	943 (84.4%)	254 (18.2%)	4,572 (48.3%)
Koranic school only	0 (0.0%)	0 (0.0%)	0 (0.0%)	236 (17.0%)	34 (2.4%)	0 (0.0%)	461 (33.0%)	731 (7.7%)
Unknown	0	3	0	12	0	0	2	17
Caregiver age (years)								
<20	161 (11.8%)	101 (7.2%)	171 (12.2%)	132 (9.4%)	56 (4.0%)	208 (18.6%)	79 (5.7%)	908 (9.6%)
20-29	857 (63.0%)	928 (66.3%)	927 (66.4%)	804 (57.5%)	879 (62.9%)	516 (46.2%)	826 (59.1%)	5,737 (60.6%)
30-39	312 (22.9%)	322 (23.0%)	278 (19.9%)	418 (29.9%)	419 (30.0%)	312 (27.9%)	423 (30.3%)	2,484 (26.2%)
>=40	31 (2.3%)	49 (3.5%)	21 (1.5%)	45 (3.2%)	44 (3.1%)	81 (7.3%)	70 (5.0%)	341 (3.6%)
Unknown	0	0	2	1	2	0	1	6
Caregiver employment status								
No formal employment	1,288 (94.6%)	703 (50.3%)	821 (58.8%)	1,331 (95.3%)	1,322 (94.4%)	967 (86.6%)	834 (59.7%)	7,266 (76.8%)
Formal employment	73 (5.4%)	695 (49.7%)	575 (41.2%)	66 (4.7%)	78 (5.6%)	150 (13.4%)	564 (40.3%)	2,201 (23.2%)
Unknown	0	2	3	3	0	0	1	9
Wealth index								
Quintile 1 (fewest assets)	0 (0.0%)	34 (2.4%)	158 (11.3%)	23 (1.6%)	458 (32.7%)	507 (45.4%)	346 (24.7%)	1,526 (16.1%)
Quintile 2	83 (6.1%)	247 (17.6%)	449 (32.1%)	430 (30.7%)	475 (33.9%)	430 (38.5%)	596 (42.6%)	2,710 (28.6%)
Quintile 3	278 (20.4%)	404 (28.9%)	538 (38.5%)	600 (42.9%)	235 (16.8%)	173 (15.5%)	355 (25.4%)	2,583 (27.3%)
Quintile 4	430 (31.6%)	522 (37.3%)	196 (14.0%)	335 (23.9%)	170 (12.1%)	7 (0.6%)	102 (7.3%)	1,762 (18.6%)
Quintile 5 (most assets)	570 (41.9%)	193 (13.8%)	58 (4.1%)	12 (0.9%)	62 (4.4%)	0 (0.0%)	0 (0.0%)	895 (9.4%)
Age-appropriate vaccination								
Has not received	270 (22.3%)	867 (71.6%)	542 (56.8%)	506 (41.2%)	527 (53.1%)	344 (36.4%)	1,044 (75.2%)	4,100 (51.7%)
Received	941 (77.7%)	344 (28.4%)	412 (43.2%)	723 (58.8%)	465 (46.9%)	602 (63.6%)	345 (24.8%)	3,832 (48.3%)
Unknown	150	189	445	171	408	171	10	1,544
Prior care-seeking								
None	487 (35.8%)	1,288 (92.0%)	1,159 (82.8%)	1,103 (78.8%)	1,228 (87.7%)	880 (78.8%)	1,314 (93.9%)	7,459 (78.7%)
Medically-attended diarrhea	447 (32.8%)	23 (1.6%)	73 (5.2%)	5 (0.4%)	155 (11.1%)	115 (10.3%)	48 (3.4%)	866 (9.1%)
Community health worker/Health outpost	70 (5.1%)	14 (1.0%)	12 (0.9%)	4 (0.3%)	8 (0.6%)	5 (0.4%)	7 (0.5%)	120 (1.3%)
Pharmacy/Drug seller	355 (26.1%)	45 (3.2%)	133 (9.5%)	186 (13.3%)	4 (0.3%)	111 (9.9%)	29 (2.1%)	863 (9.1%)
Traditional healer	2 (0.1%)	29 (2.1%)	1 (0.1%)	101 (7.2%)	5 (0.4%)	1 (0.1%)	1 (0.1%)	140 (1.5%)
Other	0 (0.0%)	1 (0.1%)	21 (1.5%)	1 (0.1%)	0 (0.0%)	5 (0.4%)	0 (0.0%)	28 (0.3%)
Pre-enrollment antibiotics received	237 (17.4%)	33 (2.4%)	54 (3.9%)	53 (3.8%)	77 (5.5%)	60 (5.4%)	42 (3.0%)	556 (5.9%)
Time to facility (minutes)	20 (15, 30)	30 (15, 40)	30 (20, 60)	15 (10, 20)	15 (10, 20)	10 (8, 20)	30 (20, 40)	20 (10, 30)
Unknown	0	4	256	10	3	2	6	281
Dehydration								
None	1,191 (87.5%)	656 (46.9%)	1,348 (96.4%)	1,236 (88.3%)	1,185 (84.6%)	214 (19.2%)	1,310 (93.6%)	7,140 (75.3%)
Some	168 (12.3%)	685 (48.9%)	49 (3.5%)	154 (11.0%)	207 (14.8%)	897 (80.3%)	70 (5.0%)	2,230 (23.5%)
Severe	2 (0.1%)	59 (4.2%)	2 (0.1%)	10 (0.7%)	8 (0.6%)	6 (0.5%)	19 (1.4%)	106 (1.1%)
Dysentery								
No	1,155 (84.9%)	1,252 (89.4%)	1,261 (90.1%)	1,307 (93.4%)	1,166 (83.3%)	947 (84.8%)	1,158 (82.8%)	8,246 (87.0%)
Yes	206 (15.1%)	148 (10.6%)	138 (9.9%)	93 (6.6%)	234 (16.7%)	170 (15.2%)	241 (17.2%)	1,230 (13.0%)
GEMS-MSD								
Less-severe	966 (71.0%)	584 (41.7%)	1,214 (86.8%)	1,161 (82.9%)	989 (70.6%)	184 (16.5%)	1,062 (75.9%)	6,160 (65.0%)
Moderate-to-severe	395 (29.0%)	816 (58.3%)	185 (13.2%)	239 (17.1%)	411 (29.4%)	933 (83.5%)	337 (24.1%)	3,316 (35.0%)
Modified Vesikari score (MVS)								
Mild	692 (50.8%)	668 (47.7%)	946 (67.6%)	1,046 (74.7%)	883 (63.1%)	456 (40.8%)	888 (63.5%)	5,579 (58.9%)
Moderate	175 (12.9%)	289 (20.6%)	235 (16.8%)	173 (12.4%)	239 (17.1%)	294 (26.3%)	248 (17.7%)	1,653 (17.4%)
Severe	494 (36.3%)	443 (31.6%)	218 (15.6%)	181 (12.9%)	278 (19.9%)	367 (32.9%)	263 (18.8%)	2,244 (23.7%)
Hospitalized during index episode								
No	1,249 (91.8%)	1,342 (95.9%)	1,398 (99.9%)	1,399 (99.9%)	1,391 (99.4%)	1,109 (99.3%)	1,345 (96.1%)	9,233 (97.4%)
Yes	112 (8.2%)	58 (4.1%)	1 (0.1%)	1 (0.1%)	9 (0.6%)	8 (0.7%)	54 (3.9%)	243 (2.6%)
Wasting								
None	1,119 (82.3%)	1,337 (95.7%)	1,327 (95.4%)	1,086 (77.6%)	913 (65.8%)	1,052 (95.5%)	1,000 (71.7%)	7,834 (83.1%)
Moderate	206 (15.1%)	48 (3.4%)	53 (3.8%)	261 (18.6%)	334 (24.1%)	44 (4.0%)	307 (22.0%)	1,253 (13.3%)
Severe	35 (2.6%)	12 (0.9%)	11 (0.8%)	53 (3.8%)	141 (10.2%)	6 (0.5%)	87 (6.2%)	345 (3.7%)
Unknown	1	3	8	0	12	15	5	44
Stunting								
Not stunted	1,029 (75.7%)	1,139 (81.6%)	988 (71.1%)	1,217 (86.9%)	844 (61.0%)	868 (78.8%)	1,106 (79.3%)	7,191 (76.3%)
Stunted	331 (24.3%)	257 (18.4%)	402 (28.9%)	183 (13.1%)	540 (39.0%)	233 (21.2%)	288 (20.7%)	2,234 (23.7%)
Unknown	1	4	9	0	16	16	5	51
Underweight								
None	1,081 (79.4%)	1,297 (92.6%)	1,224 (87.5%)	1,088 (77.7%)	799 (57.2%)	1,008 (90.2%)	919 (65.7%)	7,416 (78.3%)
Moderate	222 (16.3%)	82 (5.9%)	139 (9.9%)	239 (17.1%)	370 (26.5%)	90 (8.1%)	351 (25.1%)	1,493 (15.8%)
Severe	58 (4.3%)	21 (1.5%)	36 (2.6%)	73 (5.2%)	227 (16.3%)	19 (1.7%)	129 (9.2%)	563 (5.9%)
Unknown	0	0	0	0	4	0	0	4

^*1*^ n (%); Median (IQR).

Caregiver employment also differed substantially. Overall, 77% reported no formal employment. There was however variation across the 7 countries. 95% reported no formal employment in Mali while Kenya reported that 50% of the caregivers had no formal employment. Household wealth distribution showed Pakistan, Peru, and The Gambia had higher representation from the poorest quintile, while the wealthiest quintile was most represented in Bangladesh. Prior to enrolment, overall, 78% of caregivers reported no care seeking for the diarrhea episode of their child. Age-appropriate vaccination coverage varied across the sites. 78% of the children in Bangladesh received all age-appropriate vaccines while in Kenya only 28% of the children received age-appropriate vaccines. Overall, 24% of the children enrolled were stunted. Across the different country sites, the highest percentage of stunting was reported in children in Pakistan (39%). Overall, 83% of the children that were enrolled were not wasted, 75% of the children enrolled showed no signs of dehydration at enrollment and 87% of the children enrolled had no dysentery.

### Prevalence of delayed care-seeking

62% was the overall prevalence of delayed care seeking with some variation by site. Country-specific estimates were 78% in Bangladesh, 48% in Kenya, 50% in Malawi, 70% in Mali, 66% in Pakistan, 74% in Peru, and 51% in The Gambia. Using the sensitivity definition (≥4 days from illness onset), overall prevalence was 16% and varied from the lowest prevalence in The Gambia (5%) to the highest in Bangladesh (37%). Prevalence was 7% in Kenya, 6% in Malawi, 8% in Mali, 22% in Pakistan, and 31% in Peru

Delayed care seeking was more common among younger children, younger mothers, those who sought care prior to enrollment, and those with signs of clinical severity (Table 2). Children aged 12–23 months and 24–35 months were less likely to experience delays in care seeking compared to those aged 6–11 months, both in univariate (PR: 0.96; 95% confidence interval [95% CI]: 0.93–0.99 and PR: 0.89; 95% CI: 0.84–0.93) and multivariate models (adjusted prevalence ratio [aPR]: 0.96; 95% CI: 0.93–0.99 and aPR: 0.90; 95% CI: 0.86–0.94).

**Table 2:** Relationship between predictors and delayed care-seeking, adjusted for enrollment site.

Characteristic	Delayed care-seeking*^3^*	No delayed care-seeking*^3^*	Univariate*^1^*			Multivariate*^2^*		
			PR*^4^*	95% CI*^4^*	p-value	PR*^4^*	95% CI*^4^*	p-value
Age (months)								
6-11	2,246 / 5,848 (38%)	1,229 / 3,628 (34%)	—	—		—	—	
12-23	2,688 / 5,848 (46%)	1,684 / 3,628 (46%)	0.96	0.93, 0.99	0.009	0.96	0.93, 0.99	0.012
24-35	914 / 5,848 (16%)	715 / 3,628 (20%)	0.89	0.84, 0.93	<0.001	0.90	0.86, 0.94	<0.001
Sex								
Female	2,673 / 5,848 (46%)	1,643 / 3,628 (45%)	—	—				
Male	3,175 / 5,848 (54%)	1,985 / 3,628 (55%)	0.99	0.96, 1.02	0.463			
Maternal education								
Primary school or less	2,574 / 5,841 (44%)	1,582 / 3,618 (44%)	—	—				
Some secondary or greater	2,850 / 5,841 (49%)	1,722 / 3,618 (48%)	0.96	0.93, 1.00	0.038			
Koranic school only	417 / 5,841 (7%)	314 / 3,618 (9%)	0.98	0.91, 1.05	0.561			
Caregiver age (years)								
<20	629 / 5,843 (11%)	279 / 3,627 (8%)	—	—		—	—	
20-29	3,479 / 5,843 (60%)	2,258 / 3,627 (62%)	0.91	0.87, 0.96	<0.001	0.92	0.88, 0.96	<0.001
30-39	1,520 / 5,843 (26%)	964 / 3,627 (27%)	0.90	0.86, 0.95	<0.001	0.91	0.86, 0.95	<0.001
>=40	215 / 5,843 (4%)	126 / 3,627 (3%)	0.93	0.85, 1.02	0.115	0.92	0.84, 1.01	0.092
Caregiver employment status								
No formal employment	4,673 / 5,842 (80%)	2,593 / 3,625 (72%)	—	—				
Formal employment	1,169 / 5,842 (20%)	1,032 / 3,625 (28%)	1.00	0.95, 1.04	0.88			
Wealth index								
Quintile 1 (fewest assets)	956 / 5,848 (16%)	570 / 3,628 (16%)	—	—				
Quintile 2	1,646 / 5,848 (28%)	1,064 / 3,628 (29%)	1.01	0.96, 1.06	0.662			
Quintile 3	1,518 / 5,848 (26%)	1,065 / 3,628 (29%)	0.98	0.93, 1.04	0.569			
Quintile 4	1,115 / 5,848 (19%)	647 / 3,628 (18%)	1.04	0.98, 1.11	0.199			
Quintile 5 (most assets)	613 / 5,848 (10%)	282 / 3,628 (8%)	1.02	0.94, 1.09	0.684			
Age-appropriate vaccination								
Has not received	2,404 / 4,891 (49%)	1,696 / 3,041 (56%)	—	—				
Received	2,487 / 4,891 (51%)	1,345 / 3,041 (44%)	0.97	0.94, 1.01	0.092			
Prior care-seeking								
None	4,245 / 5,848 (73%)	3,214 / 3,628 (89%)	—	—		—	—	
Medically-attended diarrhea	717 / 5,848 (12%)	149 / 3,628 (4%)	1.32	1.26, 1.37	<0.001	1.29	1.23, 1.35	<0.001
Community health worker/Health outpost	95 / 5,848 (2%)	25 / 3,628 (1%)	1.30	1.19, 1.43	<0.001	1.25	1.13, 1.38	<0.001
Pharmacy/Drug seller	659 / 5,848 (11%)	204 / 3,628 (6%)	1.23	1.18, 1.29	<0.001	1.21	1.15, 1.27	<0.001
Traditional healer	113 / 5,848 (2%)	27 / 3,628 (1%)	1.31	1.20, 1.42	<0.001	1.29	1.19, 1.40	<0.001
Other	19 / 5,848 (0%)	9 / 3,628 (0%)	1.29	1.00, 1.67	0.047	1.23	0.97, 1.58	0.093
Pre-enrollment antibiotics received								
No	5,395 / 5,848 (92%)	3,525 / 3,628 (97%)	—	—		—	—	
Yes	453 / 5,848 (8%)	103 / 3,628 (3%)	1.22	1.17, 1.28	<0.001	1.04	0.99, 1.10	0.086
Time to facility (minutes)	20 (10, 30)	20 (15, 30)	1.00	1.00, 1.00	0.139			
Dehydration								
None	4,302 / 5,848 (74%)	2,838 / 3,628 (78%)	—	—		—	—	
Some	1,487 / 5,848 (25%)	743 / 3,628 (20%)	1.10	1.06, 1.15	<0.001	1.08	1.03, 1.13	<0.001
Severe	59 / 5,848 (1%)	47 / 3,628 (1%)	1.07	0.90, 1.28	0.435	1.03	0.87, 1.23	0.708
Dysentery								
No	4,985 / 5,848 (85%)	3,261 / 3,628 (90%)	—	—		—	—	
Yes	863 / 5,848 (15%)	367 / 3,628 (10%)	1.15	1.11, 1.20	<0.001	1.15	1.11, 1.20	<0.001
GEMS-MSD								
Less-severe	3,627 / 5,848 (62%)	2,533 / 3,628 (70%)	—	—		—	—	
Moderate-to-severe	2,221 / 5,848 (38%)	1,095 / 3,628 (30%)	1.14	1.10, 1.18	<0.001	1.13	1.09, 1.17	<0.001
Modified Vesikari score (MVS)								
Mild	3,243 / 5,848 (55%)	2,336 / 3,628 (64%)	—	—		—	—	
Moderate	1,047 / 5,848 (18%)	606 / 3,628 (17%)	1.10	1.05, 1.15	<0.001	1.08	1.04, 1.13	<0.001
Severe	1,558 / 5,848 (27%)	686 / 3,628 (19%)	1.17	1.13, 1.21	<0.001	1.13	1.09, 1.18	<0.001
Hospitalized during index episode								
No	5,692 / 5,848 (97%)	3,541 / 3,628 (98%)	—	—				
Yes	156 / 5,848 (3%)	87 / 3,628 (2%)	1.00	0.92, 1.10	0.931			
Wasting								
None	4,761 / 5,813 (82%)	3,073 / 3,619 (85%)	—	—				
Moderate	824 / 5,813 (14%)	429 / 3,619 (12%)	1.05	1.00, 1.09	0.045			
Severe	228 / 5,813 (4%)	117 / 3,619 (3%)	1.07	0.99, 1.16	0.086			
Stunting								
Not stunted	4,449 / 5,809 (77%)	2,742 / 3,616 (76%)	—	—				
Stunted	1,360 / 5,809 (23%)	874 / 3,616 (24%)	0.98	0.95, 1.02	0.353			
Underweight								
None	4,554 / 5,846 (78%)	2,862 / 3,626 (79%)	—	—				
Moderate	924 / 5,846 (16%)	569 / 3,626 (16%)	0.99	0.95, 1.03	0.637			
Severe	368 / 5,846 (6%)	195 / 3,626 (5%)	1.05	0.98, 1.12	0.149			

^*1*^ Univariate models adjusted for enrollment site.

^*2*^ Multivariate models adjusted for enrollment site and included predictors with p < 0.02 in univariate models. To avoid collinearity between severity indicators, multivariate models adjusted by dehydration and dysentery by default. Models for GEMS-MSD and MVS did not include other severity indicators.

^*3*^ n / N (%); Median (Q1, Q3).

^*4*^ PR = Prevalence Ratio, CI = Confidence Interval.

### Relationship between predictors and delayed care seeking

Caregiver age was a significant factor. Compared to caregivers under 20 years old, those aged 20–29 and 30–39 was less likely to delay seeking care in both univariate (PR: 0.91; 95% CI: 0.87–0.96 and PR: 0.90; 95% CI: 0.86–0.95) and multivariate models (aPR: 0.92; 95% CI: 0.88–0.96 and aPR: 0.91; 95% CI: 0.86–0.95). Previous care seeking showed an association with delayed presentation. Children whose caregivers previously sought care for the same illness episode were significantly more likely to present late to the study facility. In multivariate models, this included visits to medical providers (non- EFGH facility) (aPR: 1.29; 95% CI: 1.23–1.35), visits to community health workers (aPR: 1.25; 95% CI: 1.13–1.38), pharmacies or drug sellers (aPR: 1.21; 95% CI: 1.15–1.27), and traditional healers (aPR: 1.29; 95% CI: 1.19–1.40).Clinical severity indicators were also associated with delay. Children presenting with some dehydration had higher prevalence of delay (aPR: 1.08; 95% CI: 1.03–1.13), as did those with dysentery (aPR: 1.15; 95% CI: 1.11–1.20). Disease severity scores were consistent with these findings. Moderate-to-severe diarrhea (GEMS-MSD) was linked with delay (aPR: 1.13; 95% CI: 1.09–1.17), as was a moderate (aPR 1.08; 95% CI: 1.04-1.13) and severe Modified Vesikari Score (aPR: 1.13; 95% CI: 1.09–1.18). Pre-enrollment antibiotic use was also associated with delayed care seeking. In univariate analysis, children who received antibiotics prior to enrollment had a significantly higher prevalence of delayed care seeking (PR: 1.22; 95% CI: 1.17–1.28). However, this association did not remain significant in the multivariate model (aPR: 1.04; 95% CI: 0.99–1.10). Sensitivity analysis examining univariate associations among the first enrollment of each unique child found similar relationships, although the strength of the associations was attenuated (Table B in S1 Appendix).

### Relationship between predictors and very delayed care seeking

Trends were more pronounced in the sensitivity analysis (Table 3). Children aged 24–35 months were again less likely to experience very delayed care seeking (aPR: 0.85; 95% CI: 0.74–0.97) compared to infants, confirming the inverse relationship between child age and delay. Prior care seeking again showed strong associations with delay. Caregivers who first consulted medical providers (PR: 2.09; 95% CI: 1.86–2.36), community health workers (aPR: 2.10; 95% CI: 1.65–2.66), or pharmacies (aPR: 1.60; 95% CI: 1.38–1.84) had higher odds of very delayed care seeking. Visits to traditional healers were also a significant predictor (aPR: 2.06; 95% CI: 1.41–2.99). Increased delay was also associated with illness severity. Dysentery (aPR: 1.33; 95% CI: 1.19–1.48), moderate-to-severe diarrhea (aPR: 1.24; 95% CI: 1.12–1.37), and severe MVS scores (aPR: 1.43; 95% CI: 1.29–1.59) all remained significant in multivariate models. Children with moderate wasting (aPR: 1.13; 95% CI: 1.00–1.28) and severe wasting (aPR: 1.32; 95% CI: 1.07–1.63) were more likely to experience very delayed care seeking.

**Table 3:** Relationship between predictors and delayed care-seeking defined as seeking care after more than three days of illness, adjusted for enrollment site.

Characteristic	Delayed care-seeking*^3^*	No delayed care-seeking*^3^*	Univariate*^1^*			Multivariate*^2^*		
			PR*^4^*	95% CI*^4^*	p-value	PR*^4^*	95% CI*^4^*	p-value
Age (months)								
6-11	596 / 1,532 (39%)	2,879 / 7,944 (36%)	—	—		—	—	
12-23	715 / 1,532 (47%)	3,657 / 7,944 (46%)	0.95	0.87, 1.05	0.329	0.95	0.86, 1.04	0.277
24-35	221 / 1,532 (14%)	1,408 / 7,944 (18%)	0.82	0.72, 0.94	0.005	0.85	0.74, 0.97	0.018
Sex								
Female	693 / 1,532 (45%)	3,623 / 7,944 (46%)	—	—				
Male	839 / 1,532 (55%)	4,321 / 7,944 (54%)	0.99	0.91, 1.08	0.795			
Maternal education								
Primary school or less	589 / 1,531 (38%)	3,567 / 7,928 (45%)	—	—				
Some secondary or greater	893 / 1,531 (58%)	3,679 / 7,928 (46%)	1.02	0.93, 1.13	0.670			
Koranic school only	49 / 1,531 (3%)	682 / 7,928 (9%)	0.92	0.68, 1.24	0.597			
Caregiver age (years)								
<20	184 / 1,532 (12%)	724 / 7,938 (9%)	—	—				
20-29	879 / 1,532 (57%)	4,858 / 7,938 (61%)	0.88	0.77, 1.01	0.080			
30-39	412 / 1,532 (27%)	2,072 / 7,938 (26%)	0.93	0.80, 1.08	0.349			
>=40	57 / 1,532 (4%)	284 / 7,938 (4%)	0.88	0.68, 1.15	0.345			
Caregiver employment status								
No formal employment	1,314 / 1,531 (86%)	5,952 / 7,936 (75%)	—	—				
Formal employment	217 / 1,531 (14%)	1,984 / 7,936 (25%)	1.04	0.90, 1.20	0.592			
Wealth index								
Quintile 1 (fewest assets)	306 / 1,532 (20%)	1,220 / 7,944 (15%)	—	—				
Quintile 2	349 / 1,532 (23%)	2,361 / 7,944 (30%)	0.87	0.76, 1.00	0.053			
Quintile 3	336 / 1,532 (22%)	2,247 / 7,944 (28%)	0.96	0.83, 1.12	0.611			
Quintile 4	293 / 1,532 (19%)	1,469 / 7,944 (18%)	1.03	0.86, 1.23	0.738			
Quintile 5 (most assets)	248 / 1,532 (16%)	647 / 7,944 (8%)	1.03	0.85, 1.25	0.758			
Age-appropriate vaccination								
Has not received	542 / 1,296 (42%)	3,558 / 6,636 (54%)	—	—				
Received	754 / 1,296 (58%)	3,078 / 6,636 (46%)	0.94	0.85, 1.04	0.242			
Prior care-seeking								
None	880 / 1,532 (57%)	6,579 / 7,944 (83%)	—	—		—	—	
Medically-attended diarrhea	363 / 1,532 (24%)	503 / 7,944 (6%)	2.10	1.87, 2.36	<0.001	2.09	1.86, 2.36	<0.001
Community health worker/Health outpost	44 / 1,532 (3%)	76 / 7,944 (1%)	2.07	1.64, 2.62	<0.001	2.10	1.65, 2.66	<0.001
Pharmacy/Drug seller	218 / 1,532 (14%)	645 / 7,944 (8%)	1.59	1.39, 1.83	<0.001	1.60	1.38, 1.84	<0.001
Traditional healer	24 / 1,532 (2%)	116 / 7,944 (1%)	2.16	1.48, 3.14	<0.001	2.06	1.41, 2.99	<0.001
Other	3 / 1,532 (0%)	25 / 7,944 (0%)	1.19	0.45, 3.16	0.729	1.13	0.43, 2.97	0.807
Pre-enrollment antibiotics received								
No	1,354 / 1,532 (88%)	7,566 / 7,944 (95%)	—	—		—	—	
Yes	178 / 1,532 (12%)	378 / 7,944 (5%)	1.40	1.22, 1.60	<0.001	0.96	0.84, 1.11	0.595
Time to facility (minutes)	20 (10, 30)	20 (10, 30)	1.00	1.00, 1.00	0.946			
Dehydration								
None	1,030 / 1,532 (67%)	6,110 / 7,944 (77%)	—	—		—	—	
Some	489 / 1,532 (32%)	1,741 / 7,944 (22%)	1.20	1.07, 1.35	0.003	1.13	1.00, 1.27	0.050
Severe	13 / 1,532 (1%)	93 / 7,944 (1%)	1.29	0.76, 2.20	0.343	1.14	0.68, 1.93	0.617
Dysentery								
No	1,253 / 1,532 (82%)	6,993 / 7,944 (88%)	—	—		—	—	
Yes	279 / 1,532 (18%)	951 / 7,944 (12%)	1.34	1.20, 1.49	<0.001	1.33	1.19, 1.48	<0.001
GEMS-MSD								
Less-severe	828 / 1,532 (54%)	5,332 / 7,944 (67%)	—	—		—	—	
Moderate-to-severe	704 / 1,532 (46%)	2,612 / 7,944 (33%)	1.28	1.16, 1.42	<0.001	1.24	1.12, 1.37	<0.001
Modified Vesikari score (MVS)								
Mild	674 / 1,532 (44%)	4,905 / 7,944 (62%)	—	—		—	—	
Moderate	327 / 1,532 (21%)	1,326 / 7,944 (17%)	1.51	1.35, 1.70	<0.001	1.46	1.30, 1.64	<0.001
Severe	531 / 1,532 (35%)	1,713 / 7,944 (22%)	1.55	1.40, 1.72	<0.001	1.43	1.29, 1.59	<0.001
Hospitalized during index episode								
No	1,487 / 1,532 (97%)	7,746 / 7,944 (98%)	—	—				
Yes	45 / 1,532 (3%)	198 / 7,944 (2%)	0.84	0.65, 1.10	0.202			
Wasting								
None	1,218 / 1,520 (80%)	6,616 / 7,912 (84%)	—	—		—	—	
Moderate	228 / 1,520 (15%)	1,025 / 7,912 (13%)	1.15	1.02, 1.31	0.024	1.13	1.00, 1.28	0.053
Severe	74 / 1,520 (5%)	271 / 7,912 (3%)	1.39	1.12, 1.72	0.002	1.32	1.07, 1.63	0.009
Stunting								
Not stunted	1,139 / 1,520 (75%)	6,052 / 7,905 (77%)	—	—				
Stunted	381 / 1,520 (25%)	1,853 / 7,905 (23%)	0.98	0.89, 1.09	0.764			
Underweight								
None	1,169 / 1,531 (76%)	6,247 / 7,941 (79%)	—	—				
Moderate	259 / 1,531 (17%)	1,234 / 7,941 (16%)	1.09	0.97, 1.23	0.156			
Severe	103 / 1,531 (7%)	460 / 7,941 (6%)	1.12	0.93, 1.35	0.217			

^*1*^ Univariate models adjusted for enrollment site.

^*2*^ Multivariate models adjusted for enrollment site and included predictors with p < 0.02 in univariate models. To avoid collinearity between severity indicators, multivariate models adjusted by dehydration and dysentery by default. Models for GEMS-MSD and MVS did not include other severity indicators.

^*3*^ n / N (%); Median (Q1, Q3).

^*4*^ PR = Prevalence Ratio, CI = Confidence Interval.

### Differences in correlates of care seeking behaviors across study sites

There was considerable site-level variation in the predictors of delayed care seeking (Table 4). For example, older child age (24–35 months) was associated with reduced delay in Kenya (PR = 0.71; 95% CI: 0.59–0.84) and The Gambia (PR = 0.83; 95% CI: 0.70–0.98), and to a lesser extent in Bangladesh (PR = 0.90; 95% CI: 0.82–0.99), but this trend was not observed in Malawi, Pakistan, Mali, or Peru. Caregiver characteristics also showed site-specific effects. In Malawi, caregivers aged 30–39 were significantly less likely to delay care (PR = 0.75; 95% CI: 0.62–0.90) and a similar trend was seen in Pakistan (PR = 0.83; 95% CI: 0.71–0.98) and Bangladesh (PR = 0.97; 95% CI: 0.89–1.06), whereas in Kenya and Peru, there was no clear association with caregiver age. The impact of formal caregiver employment varied notably, appearing protective in Kenya (PR = 0.85; 95% CI: 0.76–0.95) and Pakistan (PR = 0.81; 95% CI: 0.65–1.00), but associated with increased delay in The Gambia (PR = 1.24; 95% CI: 1.12–1.37). Significant associations between travel time to facility in minutes and delayed care-seeking were detected in Peru and The Gambia, but the effect sizes were small (PR = 1.0004 in Peru and PR = 1.0004 in The Gambia).

**Table 4:** Relationship between predictors and delayed care-seeking by enrollment site.

Characteristic	PR (95% CI)*^1,2^*						
	Bangladesh	Kenya	Malawi	Mali	Pakistan	Peru	The Gambia
Age (months)							
6-11	—	—	—	—	—	—	—
12-23	0.94 (0.89, 1.00)	0.86 (0.77, 0.96)**	1.01 (0.90, 1.13)	0.95 (0.88, 1.02)	1.02 (0.94, 1.11)	0.96 (0.89, 1.03)	0.98 (0.87, 1.09)
24-35	0.90 (0.82, 0.99)*	0.71 (0.59, 0.84)***	0.93 (0.80, 1.08)	0.90 (0.80, 1.01)	0.97 (0.87, 1.08)	0.94 (0.84, 1.05)	0.83 (0.70, 0.98)*
Sex							
Female	—	—	—	—	—	—	—
Male	0.96 (0.90, 1.01)	1.00 (0.89, 1.12)	0.98 (0.88, 1.08)	1.03 (0.96, 1.10)	0.95 (0.88, 1.03)	1.00 (0.93, 1.08)	1.02 (0.91, 1.13)
Maternal education							
Primary school or less	—	—	—	—	—	—	—
Some secondary or greater	1.03 (0.97, 1.09)	0.90 (0.80, 1.00)	1.00 (0.90, 1.11)	1.00 (0.92, 1.08)	0.95 (0.88, 1.02)	0.91 (0.83, 0.98)*	0.90 (0.77, 1.05)
Caregiver age (years)							
<20	—	—	—	—	—	—	—
20-29	0.91 (0.84, 0.98)*	1.10 (0.88, 1.38)	0.85 (0.74, 0.98)*	0.95 (0.84, 1.06)	0.85 (0.73, 1.0)*	0.91 (0.83, 0.99)*	0.87 (0.71, 1.06)
30-39	0.97 (0.89, 1.06)	1.02 (0.79, 1.30)	0.75 (0.62, 0.90)**	0.95 (0.84, 1.07)	0.83 (0.71, 0.98)*	0.94 (0.85, 1.03)	0.85 (0.69, 1.06)
>=40	0.89 (0.72, 1.12)	1.09 (0.76, 1.55)	0.97 (0.66, 1.43)	1.04 (0.86, 1.27)	0.87 (0.67, 1.14)	0.87 (0.74, 1.03)	0.92 (0.68, 1.22)
Caregiver employment status							
No formal employment	—	—	—	—	—	—	—
Formal employment	1.01 (0.89, 1.14)	0.85 (0.76, 0.95)**	0.98 (0.88, 1.09)	0.89 (0.74, 1.07)	0.81 (0.65, 1.00)*	1.06 (0.97, 1.16)	1.24 (1.12, 1.37)***
Wealth index, simplified							
Less wealth	—	—	—	—	—	—	—
More wealth	1.20 (1.03, 1.41)*	0.97 (0.85, 1.11)	0.95 (0.85, 1.05)	0.94 (0.88, 1.01)	1.08 (1.00, 1.17)*	1.01 (0.92, 1.10)	0.95 (0.85, 1.07)
Age-appropriate vaccination							
Has not received	—	—	—	—	—	—	—
Received	1.00 (0.93, 1.07)	0.91 (0.80, 1.05)	0.91 (0.80, 1.04)	0.96 (0.89, 1.03)	0.93 (0.84, 1.02)	0.97 (0.90, 1.05)	1.11 (0.99, 1.24)
Prior care-seeking, binary							
No	—	—	—	—	—	—	—
Yes	1.22 (1.14, 1.30)***	1.33 (1.13, 1.56)***	1.30 (1.16, 1.45)***	1.22 (1.14, 1.31)***	1.26 (1.15, 1.37)***	1.27 (1.19, 1.36)***	1.75 (1.57, 1.94)***
Pre-enrollment antibiotics received							
No	—	—	—	—	—	—	—
Yes	1.10 (1.03, 1.17)**	1.64 (1.34, 2.01)***	1.35 (1.10, 1.64)**	1.14 (0.99, 1.32)	1.22 (1.08, 1.38)**	1.22 (1.10, 1.34)***	1.83 (1.63, 2.05)***
Time to facility (minutes)	1.00 (1.00, 1.00)	1.00 (1.00, 1.00)	1.00 (1.00, 1.00)	1.00 (1.00, 1.00)	1.00 (1.00, 1.00)	1.00 (1.00, 1.00)**	1.00 (1.00, 1.00)***
Dehydration							
None	—	—	—	—	—	—	—
Some	0.92 (0.83, 1.01)	1.10 (0.98, 1.23)	0.77 (0.54, 1.09)	1.06 (0.96, 1.17)	1.19 (1.09, 1.30)***	1.24 (1.11, 1.38)***	1.46 (1.25, 1.71)***
Severe	0.64 (0.16, 2.56)	1.17 (0.91, 1.50)	0.99 (0.25, 3.95)	0.74 (0.40, 1.37)	0.98 (0.57, 1.68)	1.08 (0.60, 1.93)	1.25 (0.87, 1.80)
Dysentery							
No	—	—	—	—	—	—	—
Yes	1.09 (1.02, 1.17)*	1.03 (0.86, 1.23)	1.19 (1.03, 1.39)*	1.14 (1.02, 1.28)*	1.24 (1.15, 1.35)***	1.07 (0.97, 1.17)	1.27 (1.14, 1.43)***
GEMS-MSD							
Less-severe	—	—	—	—	—	—	—
Moderate-to-severe	1.02 (0.96, 1.09)	1.09 (0.97, 1.22)	1.09 (0.95, 1.26)	1.10 (1.02, 1.20)*	1.22 (1.13, 1.31)***	1.20 (1.07, 1.35)**	1.33 (1.20, 1.47)***
Modified Vesikari score (MVS)							
Mild	—	—	—	—	—	—	—
Moderate	0.95 (0.86, 1.05)	1.10 (0.95, 1.27)	1.10 (0.96, 1.27)	0.98 (0.88, 1.09)	1.27 (1.16, 1.39)***	1.11 (1.01, 1.21)*	1.18 (1.03, 1.36)*
Severe	1.07 (1.01, 1.13)*	1.19 (1.06, 1.35)**	1.15 (1.01, 1.32)*	1.06 (0.97, 1.17)	1.28 (1.17, 1.39)***	1.13 (1.04, 1.23)**	1.43 (1.27, 1.61)***
Hospitalized during index episode							
No	—	—	—	—	—	—	—
Yes	1.00 (0.90, 1.11)	0.84 (0.61, 1.15)	0.00 (0.00, 0.00)***	1.44 (1.39, 1.49)***	1.51 (1.45, 1.57)***	1.36 (1.31, 1.41)***	0.99 (0.75, 1.31)
Wasting							
None	—	—	—	—	—	—	—
Moderate	1.00 (0.92, 1.09)	1.20 (0.93, 1.54)	0.98 (0.74, 1.30)	1.10 (1.01, 1.20)*	1.00 (0.91, 1.10)	1.02 (0.86, 1.22)	1.09 (0.96, 1.23)
Severe	1.07 (0.92, 1.24)	0.88 (0.45, 1.72)	1.09 (0.63, 1.87)	1.11 (0.95, 1.30)	1.00 (0.88, 1.14)	0.91 (0.52, 1.61)	1.21 (1.01, 1.45)*
Stunting							
Not stunted	—	—	—	—	—	—	—
Stunted	0.94 (0.88, 1.01)	0.91 (0.78, 1.06)	1.00 (0.89, 1.12)	1.01 (0.91, 1.11)	0.94 (0.87, 1.02)	1.01 (0.93, 1.10)	1.12 (0.99, 1.26)
Underweight							
None	—	—	—	—	—	—	—
Moderate	0.95 (0.87, 1.03)	1.08 (0.86, 1.36)	0.93 (0.77, 1.12)	1.05 (0.96, 1.15)	0.96 (0.88, 1.05)	1.00 (0.88, 1.14)	1.00 (0.89, 1.14)
Severe	0.92 (0.79, 1.08)	1.43 (1.05, 1.95)*	1.16 (0.88, 1.54)	1.03 (0.88, 1.20)	0.97 (0.87, 1.08)	1.00 (0.77, 1.31)	1.23 (1.06, 1.43)**
Koranic school only				0.95 (0.85, 1.05)	0.87 (0.66, 1.16)		1.02 (0.91, 1.14)

^*1*^ PR = Prevalence Ratio, CI = Confidence Interval.

^*2*^ *p < 0.05; **p < 0.01; ***p < 0.001.

Prior care seeking from informal providers was a consistent predictor of delay across all countries, with effect sizes ranging from a PR of 1.22 (95% CI: 1.14–1.30) in Bangladesh to a PR of 1.75 (95% CI: 1.57–1.94) in The Gambia, indicating that households who engaged with informal care first were likely to delay facility-based care. Pre-enrolment antibiotic use was also associated with delay in multiple settings, including Kenya (PR = 1.64; 95% CI: 1.34–2.01), Pakistan (PR = 1.22; 95% CI: 1.08–1.38), Peru (PR = 1.22; 95% CI: 1.10–1.34), and The Gambia (PR = 1.83; 95% CI: 1.63–2.05). Indicators of clinical severity such as dysentery and elevated Modified Vesikari scores were also linked to delayed care in several sites; dysentery was associated with delay in Malawi (PR = 1.19; 95% CI: 1.03–1.39), Mali (PR = 1.14; 95% CI: 1.02–1.28), Pakistan (PR = 1.24; 95% CI: 1.15–1.35), and The Gambia (PR = 1.27; 95% CI: 1.14–1.43). Similarly, severe MVS scores were significantly associated with delay in Kenya (PR = 1.19; 95% CI: 1.06–1.35), Malawi (PR = 1.15; 95% CI: 1.01–1.32), Pakistan (PR = 1.28; 95% CI: 1.17–1.39), Peru (PR = 1.13; 95% CI: 1.04–1.23), and The Gambia (PR = 1.43; 95% CI: 1.27–1.61). Wasting also demonstrated some site-level variation and was only significantly associated with increased delay in The Gambia (PR = 1.21; 95% CI: 1.01–1.45), reinforcing the importance of local context when interpreting predictors of healthcare seeking behavior.

## Discussion

In this study, we found that 61.7% of children with MAD experienced delayed healthcare seeking when applying the primary definition (≥2 days from illness onset), and 16.2% experienced very delayed health care seeking under the sensitivity definition (≥4 days). Our study identified several factors associated with delayed healthcare seeking including severe Modified Vesikari Score, dysentery, dehydration, prior informal healthcare seeking (from pharmacies, traditional healers, or community health workers), caregiver age and child age. Findings from the sensitivity analysis and from site-specific models were largely consistent with the primary analysis, supporting the robustness of the observed associations. However, stronger effect sizes were observed in the sensitivity analysis for prior care-seeking and indicators of disease severity, suggesting that these factors may play an increasingly important role among children experiencing the longest delays. This consistency across definitions strengthens confidence in our findings.

A prior study conducted in Kenya reported a rate of 68.4% delayed healthcare seeking, were delayed care seeking was defined as seeking care one or more days after the onset of a diarrhea episode [7], while a study observed in Ethiopia identified age of children, age of mothers, number of children, preference of health facilities, and marital status were factors influencing the failure to seek treatment within 24 h of recognizing diarrhea in children under the age of five [8]. These differences may be due to differences in study contexts, time periods, sample sizes, levels of community support, and the extent of health information dissemination at the community level.

The association between clinical severity and delays particularly in children presenting with dysentery or dehydration is concerning. Although these symptoms typically indicate more serious illness, they did not always prompt immediate care in our study. Similar findings were reported in Ethiopia, where it was reported that severe symptoms were sometimes outweighed by structural barriers like transport costs or long facility queues [8]. In India, a study found that caregivers often delayed healthcare when they perceived the illness as manageable without hospital intervention [9]. These findings highlight the need for strategies that combine caregiver education on danger signs with improvements to structural enablers such as transportation and affordable, accessible services.

Child age also influenced healthcare seeking patterns, children aged 12–23 months were slightly less likely to experience delayed healthcare compared to infants aged 6–11 months. This contrasts with evidence from The Gambia, where infants were prioritized for healthcare due to perceptions of greater vulnerability [10]. Such differences suggest that cultural perceptions and local norms shape healthcare seeking decisions, reinforcing the need for age inclusive health messaging and interventions tailored to community beliefs.

The association between younger caregiver age and delayed care-seeking may reflect differences in caregiving experience, health literacy, and decision-making autonomy. Younger caregivers may have less experience in recognizing symptoms requiring urgent medical attention or face greater reliance on family members when making healthcare decisions. Similarly, the observed relationship between severe clinical presentation and delayed care-seeking may indicate that symptoms progressed during prolonged periods of home management or while seeking care from informal providers before presentation to a study facility. These findings suggest opportunities to strengthen public health messaging aimed at early recognition of danger signs, particularly among younger caregivers. Interventions aimed at younger caregivers could include targeted health education during antenatal and postnatal care visits, community-based parenting support groups, and caregiver counseling focused on recognition of diarrhea danger signs. Mobile health messaging, community health worker outreach, and integration of diarrhea education into routine child health services may further improve timely care-seeking among this group. Strengthening referral networks between informal and formal healthcare providers could also reduce delays among caregivers who initially seek alternative forms of care.

The associations between diarrhea severity, prior care-seeking, and prior receipt of antibiotics and delayed care-seeking were largely consistent across site-specific models. This may indicate that these clinical factors are more universally associated with delays in care-seeking. In contrast, child age, caregiver age, and caregiver education had inconsistent associations with delayed care-seeking across sites, indicating that contextual factors such as cultural norms, underlying child population health, and healthcare systems and facilities could play a more important role in these associations. This is coherent with separate studies in Ethiopia, India, and The Gambia, which found inconsistent associations between child age, caregiver age, and caregiver education and delayed care-seeking for diarrhea ([8–10].

## Limitations

This study has several limitations. First, it relies on observational data, which limits causal inference and introduces potential biases, such as recall bias in reporting healthcare seeking behavior. Additionally, the catchment area of enrolled participants limits our ability to detect associations between travel time to facility and delayed care-seeking. Although data were collected from diverse LMIC settings, there may be unmeasured contextual factors that influence healthcare seeking decisions and were not captured in this analysis. There is also a group of children who develop diarrhea but never attended a facility and therefore are not represented in our data. Although this study was not specifically powered to identify drivers of delayed healthcare seeking for diarrhea, it includes a large and diverse sample of participants from urban and rural contexts across three continents, and we believe the findings may be generalizable to other LMIC settings.

## Conclusion

This study highlights the complex interplay of sociodemographic, clinical, and host influencing delays in healthcare seeking for childhood diarrhea in LMICs. Key predictors of delayed healthcare seeking included younger child age, prior informal healthcare seeking, and the presence of dysentery, dehydration, or severe illness. These findings underscore the urgent need for multifaceted interventions that both enhance caregiver recognition of danger signs and address access related barriers to timely care. In addition to improving health system responsiveness, there is a pressing need to expand accessible and affordable care options to ensure that financial and geographic constraints do not hinder timely treatment. Future research should further explore caregiver health literacy, trust in healthcare providers, and context-specific barriers to healthcare seeking. Understanding these underlying beliefs and decision-making processes will be critical for designing effective strategies to ensure timely care and reduce preventable child deaths. These efforts will contribute meaningfully to the achievement of Sustainable Development Goal 3 which aims to ensure healthy lives and promote well-being for all at all ages, including reducing preventable deaths among children under five years.

## Supporting information

S1 Appendix Tables A and B. **Table A:** Number of enrollments by unique child by site. Distribution of single and repeat enrolments among children enrolled in the Enterics for Global Health (EFGH)-Shigella Surveillance Study across Bangladesh, Kenya, Malawi, Mali, Pakistan, Peru, and The Gambia. **Table B:** Univariate associations between predictors and delayed care-seeking among the first enrollment for each unique child, adjusted for enrollment site. Associations between demographic, socioeconomic, clinical, and nutritional characteristics and delayed care-seeking among the first enrolment for each unique child, adjusted for enrolment site. Prevalence ratios (PRs), 95% confidence intervals (CIs), and p-values are presented.
(DOCX)

S1 Checklist Inclusivity in Global Research Checklist. Completed PLOS Inclusivity in Global Research questionnaire describing ethical approvals, community engagement activities, local researcher involvement, informed consent procedures, cultural considerations, and dissemination plans for communicating study findings to participating communities
(DOCX)
