# Supplementary material for: Drivers of delayed care-seeking among children aged 6–35 months with medically-attended diarrhea: Findings from the Enterics for Global Health (EFGH)-Shigella surveillance study, 2022–2024
Source: PLOS Glob Public Health. 2026 Nov 13;6(11):e0007433. doi: 10.1371/journal.pgph.0007433 (PMC13766290; doi:10.1371/journal.pgph.0007433)
Supplement: Supplementary file 1 [file pgph.0007433.s001.docx]

Table A. Number of enrollments by unique child by site

|  | **Number of enrollments** | | | |
| --- | --- | --- | --- | --- |
| **Enrollment site** | **1** | **2** | **3** | **4** |
| Bangladesh | 1048 | 126 | 15 | 4 |
| Kenya | 1016 | 129 | 30 | 9 |
| Malawi | 1306 | 45 | 1 | 0 |
| Mali | 1180 | 107 | 2 | 0 |
| Pakistan | 1297 | 47 | 3 | 0 |
| Peru | 864 | 97 | 13 | 5 |
| The Gambia | 1103 | 125 | 14 | 1 |

Table B. Univariate associations between predictors and delayed care-seeking among the first enrollment for each unique child, adjusted for enrollment site.

| **Characteristic** | **Delayed care-seeking***^1^* | **No delayed care-seeking***^1^* | **PR***^2^* | **95% CI***^2^* | **p-value** | |
| --- | --- | --- | --- | --- | --- | --- |
| Age (months) |  |  |  |  |  | |
| 6-11 | 2,198 / 5,323 (41%) | 1,207 / 3,264 (37%) | — | — |  | |
| 12-23 | 2,350 / 5,323 (44%) | 1,456 / 3,264 (45%) | 0.96 | 0.91, 1.02 | 0.199 | |
| 24-35 | 775 / 5,323 (15%) | 601 / 3,264 (18%) | 0.89 | 0.82, 0.97 | 0.008 | |
| Sex |  |  |  |  |  | |
| Female | 2,414 / 5,323 (45%) | 1,467 / 3,264 (45%) | — | — |  | |
| Male | 2,909 / 5,323 (55%) | 1,797 / 3,264 (55%) | 0.99 | 0.94, 1.04 | 0.696 | |
| Maternal education |  |  |  |  |  | |
| Primary school or less | 2,349 / 5,317 (44%) | 1,431 / 3,255 (44%) | — | — |  | |
| Some secondary or greater | 2,584 / 5,317 (49%) | 1,542 / 3,255 (47%) | 0.97 | 0.91, 1.03 | 0.261 | |
| Koranic school only | 384 / 5,317 (7%) | 282 / 3,255 (9%) | 0.98 | 0.87, 1.10 | 0.755 | |
| Caregiver age (years) |  |  |  |  |  | |
| <20 | 581 / 5,318 (11%) | 258 / 3,263 (8%) | — | — |  | |
| 20-29 | 3,173 / 5,318 (60%) | 2,019 / 3,263 (62%) | 0.92 | 0.84, 1.01 | 0.069 | |
| 30-39 | 1,367 / 5,318 (26%) | 873 / 3,263 (27%) | 0.90 | 0.82, 1.00 | 0.041 | |
| >=40 | 197 / 5,318 (4%) | 113 / 3,263 (3%) | 0.94 | 0.80, 1.11 | 0.453 | |
| Caregiver employment status |  |  |  |  |  | |
| No formal employment | 4,280 / 5,317 (80%) | 2,350 / 3,261 (72%) | — | — |  | |
| Formal employment | 1,037 / 5,317 (20%) | 911 / 3,261 (28%) | 1.00 | 0.93, 1.08 | 0.971 | |
| Wealth index |  |  |  |  |  | |
| Quintile 1 (fewest assets) | 885 / 5,323 (17%) | 539 / 3,264 (17%) | — | — |  | |
| Quintile 2 | 1,519 / 5,323 (29%) | 956 / 3,264 (29%) | 1.03 | 0.94, 1.12 | 0.556 | |
| Quintile 3 | 1,379 / 5,323 (26%) | 951 / 3,264 (29%) | 0.99 | 0.91, 1.09 | 0.897 | |
| Quintile 4 | 997 / 5,323 (19%) | 565 / 3,264 (17%) | 1.05 | 0.95, 1.17 | 0.348 | |
| Quintile 5 (most assets) | 543 / 5,323 (10%) | 253 / 3,264 (8%) | 1.02 | 0.90, 1.17 | 0.746 | |
| Age-appropriate vaccination |  |  |  |  |  | |
| Has not received | 2,167 / 4,428 (49%) | 1,501 / 2,723 (55%) | — | — |  | |
| Received | 2,261 / 4,428 (51%) | 1,222 / 2,723 (45%) | 0.97 | 0.91, 1.03 | 0.288 | |
| Prior care-seeking |  |  |  |  |  | |
| None | 3,874 / 5,323 (73%) | 2,883 / 3,264 (88%) | — | — |  | |
| Medically-attended diarrhea | 633 / 5,323 (12%) | 134 / 3,264 (4%) | 1.31 | 1.20, 1.44 | <0.001 | |
| Community health worker/Health outpost | 85 / 5,323 (2%) | 23 / 3,264 (1%) | 1.28 | 1.03, 1.60 | 0.026 | |
| Pharmacy/Drug seller | 605 / 5,323 (11%) | 188 / 3,264 (6%) | 1.23 | 1.12, 1.34 | <0.001 | |
| Traditional healer | 107 / 5,323 (2%) | 27 / 3,264 (1%) | 1.28 | 1.05, 1.56 | 0.014 | |
| Other | 19 / 5,323 (0%) | 9 / 3,264 (0%) | 1.28 | 0.81, 2.01 | 0.286 | |
| Pre-enrollment antibiotics received |  |  |  |  |  | |
| No | 4,899 / 5,323 (92%) | 3,166 / 3,264 (97%) | — | — |  | |
| Yes | 424 / 5,323 (8%) | 98 / 3,264 (3%) | 1.21 | 1.10, 1.34 | <0.001 | |
| Time to facility (minutes) | 20 (10, 30) | 20 (15, 30) | 1.00 | 1.00, 1.00 | 0.418 | |
| Dehydration |  |  |  |  |  | |
| None | 3,919 / 5,323 (74%) | 2,556 / 3,264 (78%) | — | — |  | |
| Some | 1,349 / 5,323 (25%) | 663 / 3,264 (20%) | 1.10 | 1.02, 1.19 | 0.016 | |
| Severe | 55 / 5,323 (1%) | 45 / 3,264 (1%) | 1.06 | 0.81, 1.38 | 0.693 | |
| Dysentery |  |  |  |  |  | |
| No | 4,560 / 5,323 (86%) | 2,942 / 3,264 (90%) | — | — |  | |
| Yes | 763 / 5,323 (14%) | 322 / 3,264 (10%) | 1.15 | 1.06, 1.24 | <0.001 | |
| GEMS-MSD |  |  |  |  |  | |
| Less-severe | 3,317 / 5,323 (62%) | 2,290 / 3,264 (70%) | — | — |  | |
| Moderate-to-severe | 2,006 / 5,323 (38%) | 974 / 3,264 (30%) | 1.14 | 1.07, 1.21 | <0.001 | |
| Modified Vesikari score (MVS) |  |  |  |  |  | |
| Mild | 2,944 / 5,323 (55%) | 2,108 / 3,264 (65%) | — | — |  | |
| Moderate | 958 / 5,323 (18%) | 536 / 3,264 (16%) | 1.11 | 1.03, 1.19 | 0.007 | |
| Severe | 1,421 / 5,323 (27%) | 620 / 3,264 (19%) | 1.17 | 1.10, 1.25 | <0.001 | |
| Hospitalized during index episode |  |  |  |  |  | |
| No | 5,175 / 5,323 (97%) | 3,184 / 3,264 (98%) | — | — |  | |
| Yes | 148 / 5,323 (3%) | 80 / 3,264 (2%) | 1.02 | 0.86, 1.20 | 0.841 | |
| Wasting |  |  |  |  |  | |
| None | 4,304 / 5,289 (81%) | 2,749 / 3,256 (84%) | — | — |  | |
| Moderate | 770 / 5,289 (15%) | 398 / 3,256 (12%) | 1.05 | 0.97, 1.13 | 0.277 | |
| Severe | 215 / 5,289 (4%) | 109 / 3,256 (3%) | 1.07 | 0.93, 1.23 | 0.374 | |
| Stunting |  |  |  |  |  | |
| Not stunted | 4,086 / 5,285 (77%) | 2,477 / 3,253 (76%) | — | — |  | |
| Stunted | 1,199 / 5,285 (23%) | 776 / 3,253 (24%) | 0.98 | 0.92, 1.04 | 0.512 | |
| Underweight |  |  |  |  |  | |
| None | 4,155 / 5,321 (78%) | 2,569 / 3,262 (79%) | — | — |  | |
| Moderate | 828 / 5,321 (16%) | 516 / 3,262 (16%) | 0.99 | 0.91, 1.06 | 0.715 | |
| Severe | 338 / 5,321 (6%) | 177 / 3,262 (5%) | 1.05 | 0.93, 1.17 | 0.443 | |
| *^1^* n / N (%); Median (Q1, Q3) *^2^* PR = Prevalence Ratio, CI = Confidence Interval | | | | | |  |
